# Gender differences in cortical morphological networks

**DOI:** 10.1007/s11682-019-00123-6

**Published:** 2019-05-17

**Authors:** Ahmed Nebli, Islem Rekik

**Affiliations:** 1 BASIRA Lab, Faculty of Computer and Informatics, Istanbul Technical University, Istanbul, Turkey; 2 Higher Institute of Applied Science and Technologies (ISSAT), University of Sousse, Sousse, Tunisia; 3 Computing, School of Science and Engineering, University of Dundee, Dundee, UK

**Keywords:** Cortical morphological networks, Gender differences, Feature selection, T1-weighted MRI, Brain connectivity, Cortical morphology, Sulcal depth

## Abstract

Cortical morphological networks (CMN), where each network models the relationship in morphology between different cortical brain regions quantified using a specific measurement (e.g., cortical thickness), have not been investigated with respect to gender differences in the human brain. Cortical processes are expected to involve complex interactions between different brain regions, univariate methods thus might overlook informative gender markers. Hence, by leveraging machine learning techniques with the potential to highlight multivariate interacting effects, we found that the most discriminative CMN connections between males and females were derived from the left hemisphere using the mean sulcal depth as measurement. However, for both left and right hemispheres, the first most discriminative morphological connection revealed across all cortical attributes involved (entorhinal cortex ↔ caudal anterior cingulate cortex) and (entorhinal cortex ↔ transverse temporal cortex) respectively, which gives us new insights into behavioral gender differences from an omics perspective and might explain why males and females learn differently.

## Introduction

The brain construct encodes subtle differences in cognitive functions between men and women. These differences emerge during foetus development period where research has shown that male fetuses appear to involute fewer overproduced cortical neurons than females (De Courten-Myers 1999). This gender difference could explain in part that the male brain undergoes greater functional impairments from early brain damage whereas the female brain exhibits a higher incidence and prevalence of dementia. Hence, since several brain disorders can be related to gender, it is important to first pin down gender differences in the healthy human brain. This can potentially help devise a personalized treatment for different neurological disorders, tailored for male and female populations, respectively. In particular, the cortex is a multi-folded complex shape nesting vital brain function and cognition. Such complexity cannot be solely grasped using low-level region to region comparison approaches across two groups. Hence, we resort to modeling the brain as a network, where the interaction between regions becomes a biological feature of interest.

A plethora of research studies investigated gender differences using brain network datasets (i.e., brain connectomics) in both health and disease based on the two most widely used measures of brain connectivity in the literature: functional connectivity and structural connectivity, derived from functional magnetic resonance imaging (fMRI) and diffusion weighted imaging (DWI). For instance, using structural neuroimaging (Gur et al. 1991; Gur and Gur 2016), found that males and females manifest different neurological patterns when it comes to aging effects on cognitive abilities. In (Gur and Gur 2017a) the authors found that aging-related changes affect more males than females. On the other hand, using structural brain networks (Ingalhalikar et al. 2014), has found that females have a strong interhemispheric connection whereas males have strong intra-hemispheric connections in most of the brain regions, which could reveal the reasons behind the observed gender-related complementarity in behavior. In a different study using functional brain networks (Gur and Gur 2017b), reported that the period between childhood and early adult shows an advancement in accuracy and speed of performance especially in executive and reasoning tasks. Another study using fMRI (Bell et al. 2006) found that males outperformed females in spatial attention tasks with males having faster reaction times. These findings were supported by (Gur and Gur 2016, 2017a) suggesting that males have better spatial processing as well as an enhanced motor speed while females have a significant word and face memory and performed better overall in social cognition tasks. Despite this growing body of research on such networks and how they encode for gender differences, however, there is still a large gap in the literature where cortical morphological networks (CMN) remain unexplored with respect to gender.

More importantly, based on the tension theory of cerebral cortex morphogenesis suggesting that cortical morphology reflects the underlying changes in the structural and functional connectome (Van Essen 1997), recent studies have started exploring morphological connections of the cortex and how they are altered by neurological disorders including dementia (Lisowska and Rekik 2018; Mahjoub et al. 2018) and autism (Soussia and Rekik 2018; Dhifallah et al. 2018), and how they are linked to cognition and genomics (Wagstyl et al. 2018). The majority of these seminal works applied machine learning on brain morphological network datasets and demonstrated their potential in unraveling the cortical brain construct from a connectional viewpoint while leveraging minimal financial resources for brain scanning without the need of costly and time-consuming fMRI and DWI. A landmark work investigated gender differences in cortical morphological complexity (Luders et al. 2004) in independent as opposed to interactive brain regions; however, no previous studies investigated how gender influences morphological connections.

The goal of this study is to investigate for the first time the most discriminative connections unveiling gender differences using cortical morphological networks as this latter approach was effective and reliable when investigating cortical connectivity fingerprinting dementia for aged patients (Lisowska and Rekik 2018).

## Materials and methods

### Overview

**Fig. 1:**
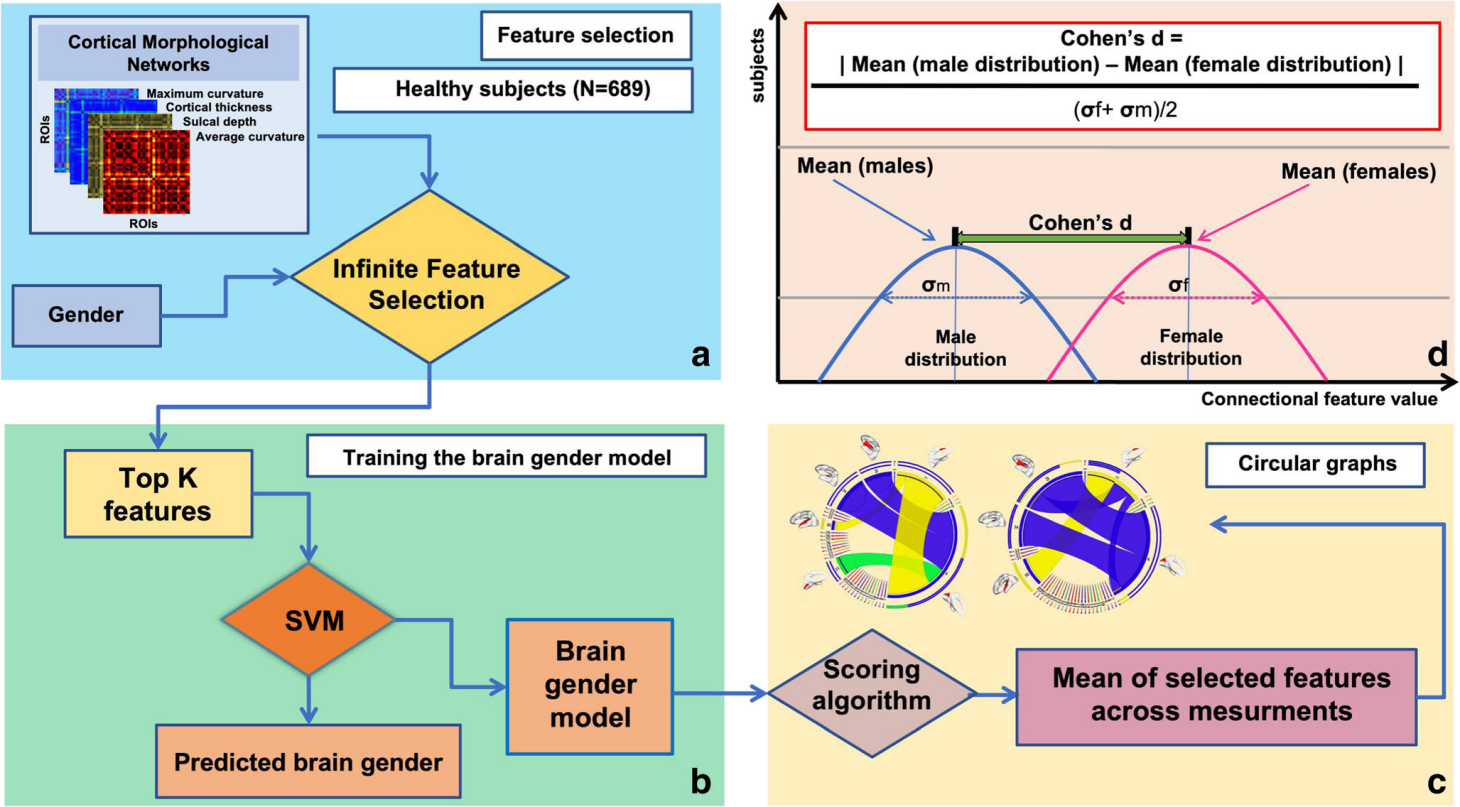
Proposed method pipeline to investigate the top connections fingerprinting gender differences. **a** We use each of the four cortical morphological networks (CMNs) encoding the similarity in morphology between different brain regions to train a supervised infinite feature selection algorithm (Roffo et al. 2015) to identify the top K most discriminative features between healthy male and female groups. **b** Next, we select the top K morphological connections derived from each CMN to train a linear classifier (support vector machine –SVM) in distinguishing between male and female cortices. **c** We devise a feature scoring algorithm by quantifying feature reproducibility across multiple cross-validation strategies (e.g., leave-one-out, 5-fold). The circular graphs display the top 5 most reproducible gender-specific cortical morphological connections across CMNs in the left and right hemispheres, respectively. **d** For each CMN, we calculate d Cohen’s coefficient of the top 5 most discriminative connections between male and female groups as detailed in Table 1

To efficiently handle the complexity of the cortical network and its multivariate interacting effects, we resort to advanced learning from data techniques which can greatly help in the extraction of truly relevant features (i.e., potential biomarkers) (Huynh-Thu et al. 2012). Such machine learning techniques can replace the original relevance score associated with a feature with a measure that can be interpreted in a statistical way and hence allow the user to determine a significance threshold in a more informed way (Huynh-Thu et al. 2012). In this study, for each CMN, we aim to identify relevant connectional features that discriminate between male and female brains. When devising a machine learning model, one needs to train and test this model. However, using the same data to train and test would not guarantee the reproducibility of the model. Thus, we need to split the data into training and testing samples. Cross-validation (CV) is a method that randomly splits available samples into training and testing sets and guarantees the best performances of the model. For reproducibility, generalizability and scalability, we deploy a multiple CV scheme including leave-one-out, 5-fold and 10-fold CV to train a support vector machines (SVM) classifier to label each CMN as either ‘male’ or ‘female’. However, the training of such classifier might be hindered by the high dimensionality of CMN features, which presents one of the major problems in machine learning. To address this issue, we leverage infinite feature selection (inFS) method (Roffo et al. 2015), where the selected connectional features are supervised by the subject gender. Finally, by selecting the top 5 highly ranked features by inFS shared across the 3 different CV schemes, we are able to highlight the most discriminative cross-validated morphological connectional features encoding gender differences for each cortical measurement. We note that SVM parameters were automatically tuned using 5-fold nested cross-validation. The number of the features selected to train SVM classifier was empirically tuned by selecting the number of features that boosted the classification across all views. We note that this does not affect the discovered most gender-discriminative features, since features are first ranked prior to SVM training. Figure 1 sketches the key steps of the proposed framework to identify the most discriminative morphological connectional features between male and female populations.

### Dataset

Our dataset is composed of 698 subjects from the Brain Genomics Superstruct Project (GSP) dataset (Buckner et al. 2012; Holmes et al. 2015), each with structural T1-w MR image, aged between 21 and 23 years old; males (n = 308; 21.6 ± 0.9 years, mean ± s.d.); females (n = 390, 21.6 ± 0.8 years, mean ± s.d.). The T1-w MRI were acquired on a Siemens head-only 3T scanner (Allegra, Siemens Medical System, Erlangen, Germany) with a circularly polarized head coil, 70 transverse slices were acquired by using a turbo spin echo (TSE) sequences: TR = 7380 nos TE = 119 mss with a Flip Angle = 150°, and resolution = 1.25 × 1.25 × 1.95*mm*^3^ (Gillmore et al. 2011). All subjects are healthy and none of them had a history of psychological or any sign of brain disorders.

**Fig. 2:**
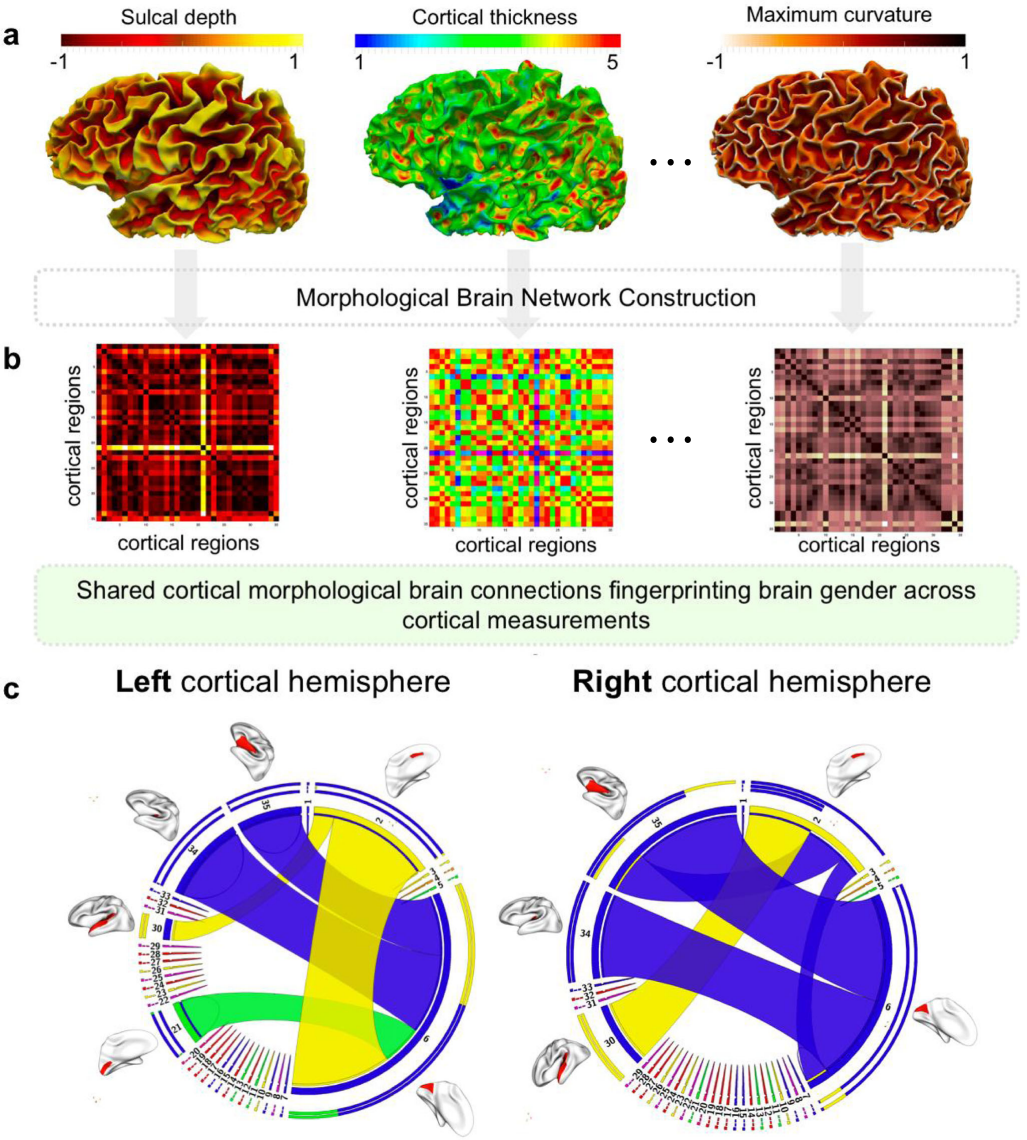
Identification of top 5 morphological cortical connections discriminating between male and female cortices in left and right hemispheres. **a** Cortical surfaces color-coded by morphological measurements (e.g., cortical thickness). **b** Cortical morphological networks derived from the cortex using different measurements. **c** Circular graphs displaying the top 5 most discriminative and cross-validated morphological connections disentangling the male from the female cortex

We examined four cortical morphological networks each derived from a specific cortical measurements including (1) maximum principal curvature, (2) cortical thickness network, (3) sulcal depth network and (4) average curvature network in a well-matched sample of healthy men (n = 308; 21.6 ± 0.9 years, mean ± s.d.) and women (n = 390; 21.6 ± 0.8 years) from the Brain Genomics Superstruct Project (GSP) (Buckner et al. 2012; Holmes et al. 2015), where participants provided written informed consent in accordance with guidelines established by the Partners Health Care Institutional Review Board and the Harvard University Committee on the Use of Human Subjects in Research. T1-weighted images were acquired using a 1.2mm isotropic resolution. Test-retest reliability was established with a correlation range from 0.75 for the estimated cortical thickness of the right medial prefrontal cortex to 0.99 for the estimated intracranial volume (Holmes et al. 2015). We used FreeSurfer processing pipeline (Fischl 2012) to reconstruct the left and right cortical hemispheres. Then we parcellated each cortical hemisphere into 35 regions using Desikan-Killiany atlas. Finally, for each subject, we used different measurements to generate a set of cortical morphological networks (CMNs) (Fig. 2b) quantifying the morphological distance in sulcal and gyral convolutions between distinct cortical regions as detailed in (Mahjoub et al. 2018).

### Data preprocessing steps

The FreeSurfer processing steps included skull stripping, motion correction, T1-w intensity normalization, topology correction and segmentation of the subcortical white matter (WM) and deep grey matter (GM) volumetric structures to identify GM/WM and GM/cerebrospinal fluid (CSF) boundaries (Dale et al. 1999). Next, following cortical hemisphere construction a topology correction, each hemisphere was parcellated into 35 anatomical regions of interest using Desikan-Killiany Atlas. For each subject, we generated *n*_*c*_ = 4 cortical morphological networks: **C**_1_ denotes the maximum principal curvature brain view, **C**_2_ denotes the mean cortical thickness brain view, **C**_3_ denotes the mean sulcal depth brain view, and **C**_4_ denotes the mean of average curvature.

### Cortical Morphological Network (CMN) definition

Following the parcellation of the cortical surface into *n*_*r*_ anatomical regions, for each *R*_*i*_ ROI and for each morphological measurement m, we compute the average cortical measurement $\overset{\sim }{\boldsymbol{m}}$across all vertices v in R as follows:

$$ {\tilde{m}}_i=\frac{1}{\#\left\{v\in {R}_i\right\}}\sum \limits_{v\in {R}_i}m(v), $$

where # {*v* ∈ *R*_*i*_} denotes the number of vertices v belonging to ROI *R*_*i*_ and m(v) the cortical measurement value assigned to vertex v. To define the morphological connection **C**_*m*_(*i*, *j*)in network **C**_*m*_ between ROIs *R*_*i*_ and *R*_*j*_ , we compute the absolute distance between averaged cortical measurement in both ROIs: $\left|{\overset{\sim }{\boldsymbol{m}}}_i-{\overset{\sim }{\boldsymbol{m}}}_j\right|$. Given *n*_*r*_ cortical regions in each hemisphere, the size of each fully connected morphological network *n*_*r*_ × *n*_*r*_ . We note that according to our definition, as two ROIs *R*_*i*_ and *R*_*j*_ become similar in morphology, their morphological connectivity **C**_*m*_(*i*, *j*) tends to 0. For each subject s, we define a feature vector ${\mathbf{f}}_m^s$ using measurement *m*. Basically, since each CMN is symmetric, we extract the off-diagonal elements of its upper triangular part. The dimension of each feature vector is thus equal to *n*_*f*_ = *n*_*r*_ × (*n*_*r*_ − 1)/2 . For *n*_*r*_ = 35, each cortical hemisphere is then represented by 595-dimentional feature vector.

### Supervised gender-related feature selection

Supervised feature selection methods allow to reduce the dimensionality of the training feature vectors by learning how to select the optimal discriminative set of features supervised by the target sample label (in our case male and female labels), thereby eliminating redundancy and irrelevant features. In this study, we leverage supervised inFS method (Roffo et al. 2015), which has several appealing aspects. For instance, while typical feature selection methods evaluate and rank features individually, inFS accounts for potential interactions among features by (i) modeling the relationship between features, then (ii) assigning a significance score to each feature by taking into account all the possible feature subsets as paths connecting them. To do so, we first define a set $\mathcal{F}$of feature distributions $\mathcal{F}=\left\{{\mathcal{N}}_1\left({\mu}_1,{\sigma}_1\right),\dots, {\mathcal{N}}_{n_f}\left({\mu}_{n_f},{\sigma}_{n_f}\right)\right\}$, where *μ* and *σ* respectively denote the rank and the weight of the corresponding feature. Next, $\mathcal{F}$is used to define an adjacency $\mathrm{matrix}\ \mathbf{A}\in {\mathbb{R}}^{n_f\times {n}_f}$ modeling the relationship in statistical distribution between all pairs of features. An element **A**(*k*, *l*) quantifying the statistical relationship between features *k* and *l* is defined as: **A**(*k*, *l*) = *ασ*_*kl*_ + (1 − *α*)*c*_*kl*_, where *α* is a loading coefficient (*α* ∈ [0, 1]), *σ*_*k*, *l*_ =  *max* (*σ*^(*k*)^, *σ*^(*l*)^) with *σ*^(*i*)^ being the standard deviation over samples of feature *f*^(*i*)^ and *c*_*kl*_ = 1 − |*Spearman* (*f*^(*i*)^, *f*^(*j*)^) | with *Spearman* being the Spearman’s rank correlation coefficient. Last, **A** is used to score each feature according to its relevance to the target discriminative task as follows: $\tilde{s}={\left[\tilde{\mathbf{S}}\mathbf{e}\right]}_i$, where ***e*** is an array of ones and$\tilde{S}={\left(\mathbf{I}-r\mathbf{A}\right)}^{-1}-\mathbf{I}$. We note that **I** represents the identity matrix and *r* a real-valued regularization factor set to 0.01.

### Supervised classifier learning

Following the ranking of the most discriminative features between both classes (i.e., male and female brains), we select the top ***K*** = 100 features to train an SVM classifier using leave-one-out (LOO) cross-validation strategy. It is important to note that cross-validation ensures the independence between feature selection and classification steps, thereby eliminating spurious effects and incorrect population-level inferences (Vul et al. 2009). From the standpoint of scientific rigor, cross-validation is a more conservative way to infer the presence of a gender-CMN relationship than is correlation. Cross-validation is designed to protect against overfitting by testing the strength of the relationship in a novel (unseen) sample, increasing the likelihood of replication in future studies. Testing and reporting performance in independent samples will facilitate evaluation of the generalizability of neuroscientific findings (Shen et al. 2017). Given *n* subjects, LOO CV learns the classifier model using (*n* − 1) training samples and their corresponding labels (male or female), then tests the learned model on the left-out subject to predict its gender. This process is iterated n times.

### Multiple cross-validations for model reproducibility and scalability

For rigorous scalability and reproducibility, we use three different cross-validation techniques to identify gender-related connectional features: LOO, 5-fold, and 10-fold. The 5-fold CV techniques are based on the same approach of the LOO technique, except that instead of taking only one sample for testing and do the iterations (which may take a certain amount of time especially for a large dataset), we split the data into *n*_*cv*_ = 5, wherein each iteration we train the classifier which ( *n*_*cv*_ − 1) subsets, test the performances with the rest one and continue the iterations until we have used all subsets for training and testing. The same strategy is deployed for 10-F CV where *n*_*cv*_ = 10.

### Identification of gender discriminative connectional features

The feature selection step allows removing the non-relevant and redundant connectional features by assigning an importance weight to each feature and ranking it according to its relevance to gender. Next, we only select the top k_f_ features with the highest ranks. For each cross-validation strategy, we obtain a feature ranking vector and its associated weight vector. Next, we extract the top k_f_ discriminative connections revealed by each deployed cross-validation scheme. Next, for each cortical measurement, we compute the average weights of top k_f_ features across the three CV strategies. We note that this operation could result in finding ${\mathrm{k}}_{{\mathrm{f}}^{\prime }}>{\mathrm{k}}_{\mathrm{f}}$ due to different top k_f_ features for each cross-validation strategy. Last, we average the latter weights across measurements and we select only the top k_f_ features. The circular graph in Fig. 2c displays the top k_f_ = 5 discriminative connectional features.

### Statistics

To evaluate the significance of our findings, we calculate the Cohen’s d index which indicates the standardized difference between two sets of data points (male and female CMNs in our case). For each identified top discriminative connectional feature connection ROIs *R*_*i*_ and *R*_*j*_, we estimate its mean *μ*_*f*_(*i*, *j*) and standard deviation *σ*_*f*_(*i*, *j*) in the female group (resp., *μ*_*m*_(*i*, *j*) and *σ*_*m*_(*i*, *j*) in the male group). *d*(*i*, *j*) is then defined as:

$$ \frac{\mid {\mu}_m\left(i,j\right)-{\mu}_f\left(i,j\right)\mid }{stdm}, where\ stdm=\frac{\sigma_m\left(i,j\right)+{\sigma}_f\left(i,j\right)}{2}. $$

We note that Cohen’s d is a number between 0 and 1. The higher the d index (i.e., closer to 1) the higher the effect is.

## Results and discussion

### Classification results revealing most gender-discriminative CMN

**Fig. 3:**
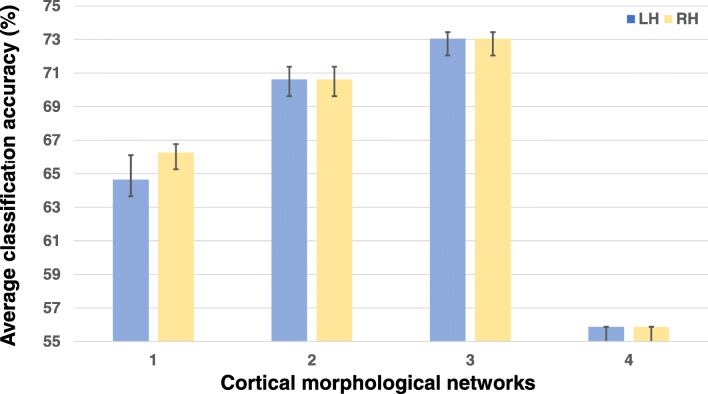
Gender classification accuracy for the left and the right hemispheres (LH and RH). Four cortical measurements were used: (1) maximum principal curvature, (2) cortical thickness network, (3) sulcal depth network and (4) average curvature network. We report the average classification accuracy across four different cross-validation strategies: leave-one-out, 5-fold and 10-fold using each cortical measurement

Figure 3 shows that SVM classifier achieved the best classification accuracy results when predicting gender from sulcal depth in both left and right hemispheres with a slightly better performance in the left hemisphere (Cohen’s coefficient d = 0.99 in Table 1). This might indicate that this cortical attribute holds the most discriminative gender differences connectivities. For instance (Gur and Gur 2017a), found that females have a better memory speed and accuracy while (Im et al. 2008) found that sulcal depth plays a major role in memory construction, which might give insights into gender differences.

**Table 1:**
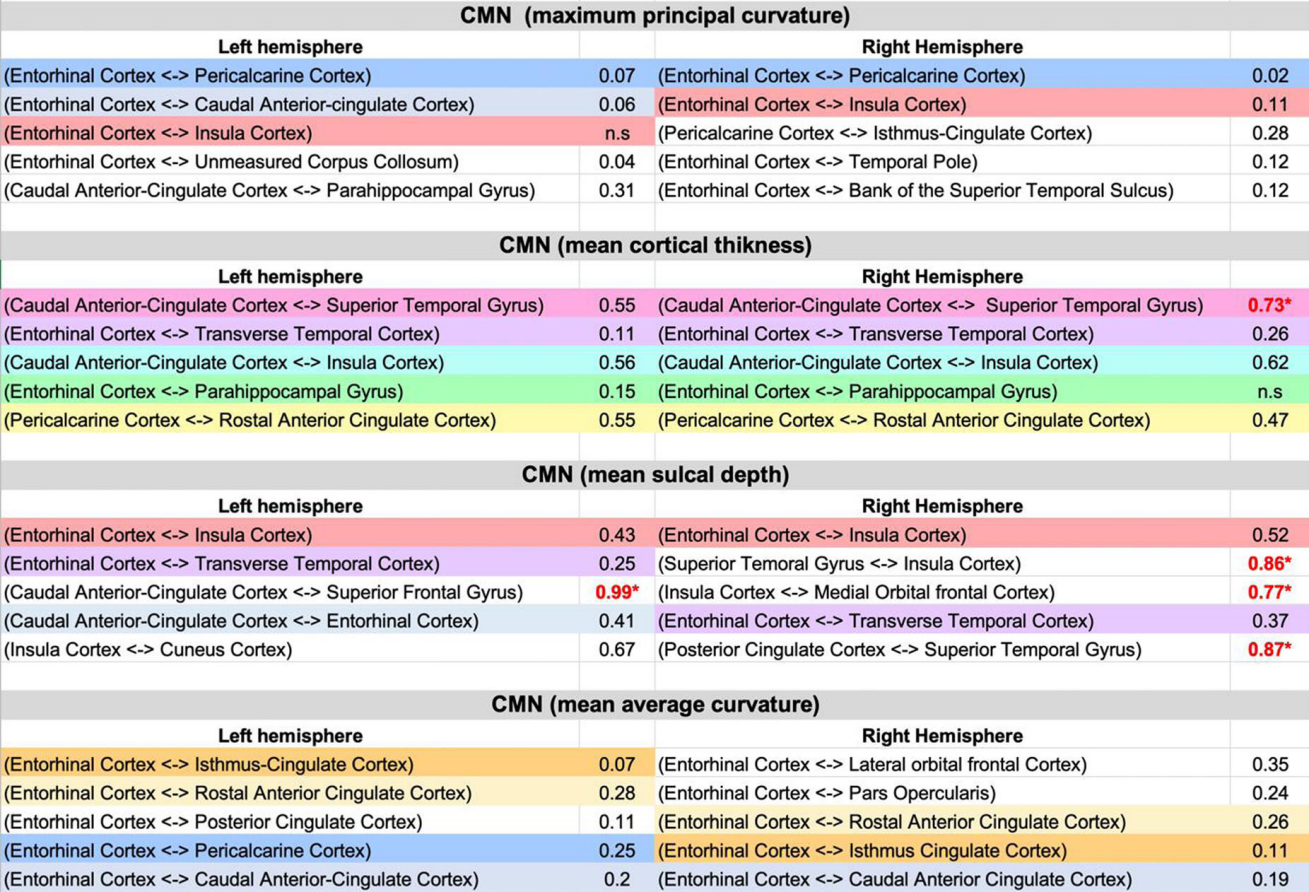
Most discriminative morphological connections revealed using cortical morphological brain networks and statistics

According to Cohen (1988), an effect size of d = 0.8 constitutes a large effect (**bold**), d = 0.5 a medium effect (italic), and d = 0.2 a small effect. (*) Regions with significant differences between men and women. n.s., differences not significant between males and females. Brain connections that were reproduced across cortical measurements are colored.

To the best of our knowledge, while no study had investigated the role of the left-hemispheric sulcal depth in gender differences (Tian et al. 2011), found that males and females are both globally efficient in their right hemisphere but females are more locally efficient in their left hemisphere, which indicates strong gender-related differences in the left hemisphere.

### Cohen’s d results revealing most gender-discriminative CMN

Capitalizing on 698 healthy individuals (390 females and 308 males) all aged between 21 and 23 years old and by selecting the top 5 highly ranked features derived from inFS shared across the 3 different CV schemes, we identified the most discriminative cross-validated morphological connectional features encoding gender differences for each cortical measurement as shown in Table 1. In fact, with a *d* = 0.99, the morphological connection between the caudal anterior cingulate cortex and the superior frontal gyrus in the left hemisphere was identified as most discriminative between male and female cortices, which is in line with the classification accuracy results by SVM classifier in (Fig. 3). Moreover, the connection between the posterior cingulate cortex and the superior temporal gyrus scored second with *d* = 0.87 and the one between the superior temporal gyrus and the insular cortex scored third *d* = 0.86. The morphological connection between the medial orbital frontal cortex and the insular cortex had a d coefficient of *d* = 0.77 and the fifth most discriminative cortical morphological connection was established between the caudal anterior cingulate cortex and the superior temporal gyrus with *d* = 0.73. We found that the top four of these top five most discriminative connections were derived from the sulcal depth, which was shown to be the best cortical attribute for gender prediction as displayed in (Fig. 3).

### Insights into top 5 most discriminative cortical morphological connections

(Singer et al. 2009) found that insula cortex is highly engaged in emotional processing (Jabbi et al. 2007; Saarela et al. 2006; Singer et al. 2004, 2006, 2008). Found that insula cortex (in particular its anterior part is known as anterior insula) had a significant role in empathy and emotional processing such as testing pleasant/ unpleasant drinks as reported in (Jabbi et al. 2007). These findings are well supported by the fact that the insular cortex is anatomically related to the amygdala (Diano et al. 2017), known as one of the highest performance emotional processors in the human brain. Another study led by (Chang et al. 2010) found that the superior temporal gyrus has a relevant role in phonetic processing due to its high speech responsiveness and thus supporting the belief that the superior temporal gyrus is a high-performance language processor.

Furthermore, as reported in (Boisgueheneuc et al. 2006), the left superior frontal gyrus is found to be having a significant role in the construction of the working memory neural network. Moreover, the caudal anterior cingulate cortex is widely known to be involved in the sensory-motor (Naito et al. 2000), found that this region is highly involved in the motor reactions guaranteeing the speed of these letters. In another study (Addis et al. 2007), found that the posterior cingulate cortex is active when people retrieve their autobiographical memories or plan for the future, this could lead the thought that the posterior cingulate cortex is engaged in the long-term memory activities.

Therefore, characterizing the connectivity between the caudal anterior cingulate cortex and the superior frontal gyrus in the left hemisphere as a gender differences biomarker is backed in literature supporting that males outdo females in the motor tasks and have a better working memory. Additionally, the connectivity between the superior temporal gyrus and posterior cingulate cortex justify why females have higher results in verbal and memory tasks comparing the males (Gur and Gur 2016; Hedges and Nowell 1995). Finally, the connectivity linking the superior temporal gyrus to the insular cortex is thought to be a significant gender differences biomarker where females are known to score better results in emotional and speech processing.

For each cortical hemisphere, we compared different CMNs between males and females and examined their interactions with gender. For each cortical measurement, Table 1 displays the top 5 most discriminative morphological brain connections consistently revealed by our three cross-validated analyses. Using maximum principal curvature measurement, the most relevant connection for both hemispheres connected the entorhinal cortex and the pericalcarine cortex. Using cortical thickness, the most gender discriminative connection linked the caudal anterior cingulate cortex with the superior temporal gyrus for both hemispheres. Using sulcal depth, we found that the connection between the entorhinal cortex and insula cortex was most discriminative. As for the average curvature measurement, the left entorhinal cortex and isthmus cingulate cortex connection and the right entorhinal cortex and lateral orbital frontal cortex were identified as gender discriminative. Figure 2 displays the top 5 most discriminative connections between male and female brains averaged across all four cortical measurements. Interestingly, for both hemispheres, we found that the entorhinal cortex acts as a morphological ‘hub’ in CMNs derived from measurements 1,2 and 4 whereas the caudal anterior cingulate cortex acts as a hub in CMNs constructed using sulcal depth.

Given the results we found when searching for the top 5 discriminative connectivities differencing males from females, both entorhinal cortex and the caudal anterior cingulate cortex acted as morphological connectional hubs. We also found a recurrent pattern wherein most of the top discriminative connectivities involved memory-related regions linking to either motor related or emotionally related regions. Knowing that females are better in emotional and speech processing and males are better in motor tasks we can conclude three major results: (i) given the fact that memory is highly engaged in the learning process, we found that males and females do learn in different ways: males learn more through motor experiences while females learn more from emotional experiences which lead us to (ii): consider that ovarian hormones play a major role in shaping emotional processing and thus the memorization and learning process in females, the absence of these hormones after menopause could justify why females are more prone to dementia and other neurodegenerative diseases (Truzzi et al. 2012), and (iii) some of the top discriminative connectivities are slightly different between hemispheres, which justifies the human brain connectional asymmetry (McGlone 1980). Our results are in line with the literature and could explain several behavioral and physiological findings.

These findings might be behaviorally and thus politically interpreted in a way to solve the long-lasting debated laws to achieve gender equity. There are some perceptions that gender differences can give justifications to the wide variety of proposed theories suggesting that gender equality does not necessarily guarantee gender equity, that females and males need different treatments especially when it comes to addressing psychological disorders and that males and females need to be taught differently.

However, to sustain the scientific transparency, some limitations need to be spotted. On one hand, this study was performed on a narrow age range (all the subjects were aged between 21 and 23 years old). It was also conducted without any backed up behavioral experiences. We intend to investigate the link between the gender discriminative morphological connectivities in relation to behavior in our future work.

## Conclusion

In this paper, we presented the first study to investigate the connectional morphology of the cortex responsible for gender differences using cortical morphological networks derived from a population of 698 individuals. We proposed a gender classification framework which leverages a landmark feature selection method. By identifying the reproduced connections across different cross-validation strategies, we found that cortical morphological connections involving (entorhinal cortex ↔ caudal anterior cingulate cortex) and (entorhinal cortex ↔ transverse temporal cortex) acted as most discriminative connections fingerprinting gender differences. These are located in memory-related regions linked to either emotional processing or motor processing. Cortical morphological networks are a nascent connectional representation of the brain connectome, which can give unprecedented insights into gender differences in relation to behavior, learning, and cognition. In our future work, we will investigate gender-behavior relationship using CMNs in both healthy and disordered populations.

## Data Availability

The open access Brain Genomics Superstruct Project (GSP) (Buckner et al. 2012; Holmes et al. 2015) data that support the findings of this study are available from https://www.nitrc.org/projects/gspdata website. For reproducibility and comparability the authors will make available upon request all morphological networks generated based on the four cortical attributes (maximum principal curvature, cortical thickness, sulcal depth, and average curvature) for 698 subjects (308 men and 390 women) following the signed approval by GSP Consortium. The machine learning MATLAB code for classification and discriminative cortical connection identification is also available from the authors upon request.

## References

[CR1] Addis DR, Wong AT, Schacter DL (2007). Remembering the past and imagining the future: Common and distinct neural substrates during event construction and elaboration. Neuropsychologic.

[CR2] Bell EC, Willson MC, Wilman AH, Dave S, Silverstone PH (2006). Males and females differ in brain activation during cognitive tasks. Neuroimage.

[CR3] Boisgueheneuc FD, Levy R, Volle E, Seassau M, Duffau H, Kinkingnehun S, Samson Y, Zhang S, Dubois B (2006). Functions of the left superior frontal gyrus in humans: a lesion study. Brain.

[CR4] R. Buckner, M. Hollinshead, A. Holmes, D. Brohawn, J. Fagerness, T. OKeefe, J. Roffman, The brain genomics superstruct project, Harvard Dataverse Network (2012).

[CR5] Chang EF, Rieger JW, Johnson K, Berger MS, Barbaro NM, Knight RT (2010). Categorical speech representation in human superior temporal gyrus. Nature Neuroscience.

[CR6] Cohen, J. (1988) Statistical power analysis for the behaviors science. 2nd. New Jersey: Laurence Erlbaum Associates, Publishers, Hillsdale

[CR7] Dale AM, Fischl B, Sereno MI (1999). Cortical surface-based analysis: I. segmentation and surface reconstruction. Neuroimage.

[CR8] De Courten-Myers GM (1999). The human cerebral cortex: Gender differences in structure and function. Journal of Neuropathology and Experimental Neurology.

[CR9] Dhifallah, S., Rekik, I., & Alzheimer's Disease Neuroimaging Initiative. (2019). Clustering-based multi-view network fusion for estimating brain networkatlases of healthy and disordered populations.* Journal of Neuroscience Methods, 311* 426–435 10.1016/j.jneumeth.2018.09.028 30282004

[CR10] Diano M, Tamietto M, Celeghin A, Weiskrantz L, Tatu MK, Bagnis A, Duca S, Geminiani G, Cauda F, Costa T (2017). Dynamic changes in amygdala psychophysiological connectivity reveal distinct neural networks for facial expressions of basic emotions. Scientific Reports.

[CR11] Fischl B (2012). Freesurfer. Neuroimage.

[CR12] Gillmore R, Stuart S, Kirkwood A, Hameeduddin A, Woodward N, Broughs AK, Meyer T (2011). Easl and mrecist responses are independent prognostic factors for survival in hepatocellular cancer patients treated with transarterial embolization. Journal of Hepatology.

[CR13] Gur RE, Gur RC (2016). Sex differences in brain and behavior in adolescence: Findings from the Philadelphia neurodevelopmental cohort. Neuroscience and Biobehavioral Reviews.

[CR14] Gur RC, Gur RE (2017). Complementarity of sex differences in brain and behavior: From laeality to multimodal neuroimaging. Journal of Neuroscience Research.

[CR15] Gur RC, Gur RE (2017). Complementarity of sex differences in brain and behavior: From laterality to multimodal neuroimaging. Journal of Neuroscience Research.

[CR16] Gur RC, Mozley PD, Resnick SM, Gottlieb GL, Kohn M, Zimmerman R, Heman G, Atlas S, Grossman R, Berretta D (1991). Gender differences in age effect on brain atrophy measured by magnetic resonance imaging. Proceedings of the National Academy of Sciences.

[CR17] Hedges LV, Nowell A (1995). Sex differences in mental test scores, variability, and numbers of high-scoring individuals. Science.

[CR18] Holmes AJ, Hollinshead MO, OKeefe TM, Petrov VI, Fariello GR, Wald LL, Fischl B, Rosen BR, Mair RW, Roffman JL (2015). Brain genomics superstruct project initial data release with structural, functional, and behavioral measures. Scientific Data.

[CR19] Huynh-Thu VA, Saeys Y, Wehenkel L, Geurts P (2012). Statistical interpretation of machine learning-based feature importance scores for biomarker discovery. Bioinformatics.

[CR20] Im K, Lee JM, Seo SW, Kim SH, Kim SI, Na DL (2008). Sulcal morphology changes and their relationship with cortical thickness and gyral white matter volume in mild cognitive impairment and alzheimer’s disease. Neuroimage.

[CR21] Ingalhalikar M, Smith A, Parker D, Satterthwaite TD, Elliott MA, Ruparel K, Hakonarson H, Gur RE, Gur RC, Verma R (2014). Sex differences in the structural connectome of the human brain. Proceedings of the National Academy of Sciences.

[CR22] Jabbi M, Swart M, Keysers C (2007). Empathy for positive and negative emotions in the gustatory cortex. Neuroimage.

[CR23] Lisowska, A., Rekik, I., AbbVie, Alzheimer's Association, Alzheimer's Drug Discovery Foundation, Araclon Biotech, Bio-Clinica, Inc., ... & Eisai, Inc.(2018). Joint pairing and structured mapping of convolutional brain morphological multiplexes for early dementia diagnosis.*Brain connectivity, 9*,(1), 22-36. 10.1089/brain.2018.0578 PMC6909728 29926746

[CR24] Luders E, Narr KL, Thompson PM, Rex DE, Jancke L, Steinmetz H, Toga AW (2004). Gender differences in cortical complexity. Nature Neuroscience.

[CR25] Mahjoub I, Mahjoub MA, Rekik I (2018). Brain multiplexes reveal morphological connectional biomarkers fingerprinting late brain dementia states. Scientific Reports.

[CR26] McGlone J (1980). Sex differences in human brain asymmetry: A critical survey. Behavioral and Brain Sciences.

[CR27] Naito E, Kinomura S, Geyer S, Kawashima R, Roland PE, Zilles K (2000). Fast reaction to different sensory modalities activates common fields in the motor areas, but the anterior cingulate cortex is involved in the speed of reaction. Journal of Neurophysiology.

[CR28] Roffo, G., Melzi, S., Castellani, U., & Vinciarelli, A. (2017). Infinite latent feature selection: a probabilistic latent graph-based ranking approach. *In Proceedings of the IEEE International Conference on Computer Vision*, (pp 1398–1406)

[CR29] Saarela, M. V., Hlushchuk, Y., Williams, A. C. D. C., Schürmann, M., Kalso, E., & Hari, R. (2006). The compassionate brain: humans detect intensity ofpain from another's face. *Cerebral Cortex, 17*(1), 230–237. 10.1093/cercor/bhj141 16495434

[CR30] Shen X, Finn ES, Scheinost D, Rosenberg MD, Chun MM, Papademetris X, Constable RT (2017). Using connectome-based predictive modeling to predict individual behavior from brain connectivity. Nature Protocols.

[CR31] Singer T, Seymour B, O’doherty J, Kaube H, Dolan RJ, Frith CD (2004). Empathy for pain involves the affective but not sensory components of pain. Science.

[CR32] Singer T, Seymour B, O’doherty JP, Stephan KE, Dolan RJ, Frith CD (2006). Empathic neural responses are modulated by the perceived fairness of others. Nature.

[CR33] Singer T, Snozzi R, Bird G, Petrovic P, Silani G, Heinrichs M, Dolan RJ (2008). Effects of oxytocin and prosocial behavior on brain responses to direct and vicariously experienced pain. Emotion.

[CR34] Singer T, Critchley HD, Preuscho↵ K (2009). A common role of insula in feelings, empathy and uncertainty. Trends in Cognitive Sciences.

[CR35] Soussia M, Rekik I (2018). Unsupervised manifold learning using high-order morphological brain networks derived from t1-w mri for autism diagnosis. Frontiers in Neuroinformatics.

[CR36] Tian L, Wang J, Yan C, He Y (2011). Hemisphere and gender-related differences in small-world brain networks: A resting-state functional mri study. Neuroimage.

[CR37] Truzzi A (2012). Burnout in familial caregivers of patients with demetia. Brazilian Journal of Psychiatry.

[CR38] Van Essen DC (1997). A tension-based theory of morphogenesis and compact wiring in the central nervous system. Nature.

[CR39] Vul E, Harris C, Winkielman P, Pashler H (2009). Puzzlingly high correlations in fmri studies of emotion, personality, and social cognition. Perspectives on Psychological Science.

[CR40] Wagstyl K, Reardon PK, Clasen L, Liu S, Messinger A, Leopold DA, Bullmore ET (2018). Morphometric similarity networks detect microscale cortical organization and predict interindividual cognitive variation. Neuron.

